# Public Health Strategies for the Gradual Lifting of the Public Sector Lockdown in Jordan and the United Arab Emirates During the COVID-19 Crisis

**DOI:** 10.2196/20478

**Published:** 2020-07-21

**Authors:** Raeda AlQutob, Immanuel Azaad Moonesar, Mohammad Rasoul Tarawneh, Mohannad Al Nsour, Yousef Khader

**Affiliations:** 1 Department of Family and Community Medicine University of Jordan Amman Jordan; 2 Health Policy/Academic Affairs Mohammed Bin Rashid School of Government Dubai United Arab Emirates; 3 Global Health Development Amman Jordan; 4 Department of Public Health Jordan University of Science and Technology Irbid Jordan

**Keywords:** COVID-19, health policies, lockdown, recovery, exit strategy, public sector, Jordan, United Arab Emirates

## Abstract

In this viewpoint, we present public policies and public health strategies for a gradual lockdown lifting during the coronavirus disease (COVID-19) crisis in two country cases, Jordan and the United Arab Emirates. While managing pandemics is critical in terms of preparedness, response, and recovery, it is equally vital to ensure that the measures for a lockdown exit are both efficient and effective. It is critical to learn from first-wave lessons to systematize responses during times of crisis and execute appropriate public policies and public health strategies. This viewpoint highlights the importance of the following during lockdown lifting: pandemic control, health care capacity, training, scaling up of resources and systems, and priority setting of public policies by acknowledging challenges, developing policy insights, and setting the policy direction. The systematic approaches and leadership thinking required for lifting lockdowns during a crisis include the three Rs: *Readiness, Responses,* and *Resilience & Recovery.*

## Introduction

About 5 million people have been infected with coronavirus disease (COVID-19) worldwide, with over 324,000 deaths as of May 20, 2020 [1]. According to the latest World Health Organization (WHO) situation report, the United Arab Emirates (UAE) has entered the community transmission phase of the pandemic with 25,063 confirmed cases and 227 deaths as of May 20, 2020, while Jordan has contained clusters of cases with 649 confirmed and 9 deaths [1]. The overall goal while lifting the lockdown is a continued reduction in the incidence of COVID-19 cases in the absence of a pharmaceutical intervention such as vaccine and medical treatment. It is critical to learn from first-wave experiences to systematize responses during times of crisis. As the situation continues to evolve, public policies will similarly have to adapt to accommodate and mitigate this change and better serve their purpose of protecting the well-being of the population [2]. Reorienting health system priorities and public sector systems to be proactive, preventive, and protective [2-4] will allow us to stay ahead of the curve, not just attempt to flatten it. Most countries, including Jordan and the UAE, have been recently moving from the “response” phase of epidemic management to the “recovery” phase; therefore, one of the many strategies for consideration is the lifting of the public sector lockdown. The approach of this viewpoint on lockdown exit strategies and recommendations for public sector institutions is based on a review of current practices, initiatives, and studies in Jordan and the UAE.

## The COVID-19 Situation in Jordan and the UAE

According to the Ministry of Health in Jordan, the first confirmed case was reported on March 2, 2020 [5]. The number of cases suddenly increased to 8 starting on March 15 and has been rising since then. According to the World Health Organization (WHO) Situation Report #83 released on April 12, 2020, Jordan was classified as having a “cluster of cases” transmission for the severe acute respiratory syndrome coronavirus 2 (SARS-CoV-2) [6]. To control this imminent threat, Jordan has enforced infection prevention and control measures and activated the National Epidemiology Committee. As of March 17, 2020, the government called for social distancing, halted all forms of inbound and outbound movement/ international travel, and enacted the Defence Law, which transferred the authority to the Minister of Defence to work and formulate orders in response to the situation [3,4]. The National Crises Management Center in coordination with government bodies took over the enforcement, follow-up, and implementation of the Defence Law orders. Consequently, a national curfew was ordered to ensure complete country isolation [3,4]. It also ordered a lockdown on all border arrivals to the country before March 17 from pandemic countries, and administrative governorates were isolated from each other. Awareness messages were targeted at children and adults older than 60 years [3,4]; they were placed under strict stay-at-home measures and their caretakers were not permitted to accompany them outside the home for any reason except in an emergency. Confirmed and suspected COVID-19 cases from airport arrivals by March 17 were isolated in hospitals under the strict supervision of qualified medical staff [3,4]. Moreover, the government immediately took measures to ensure the preparedness of the health sector. Instantly, equipment and supplies necessary for diagnosis were ordered and put at the disposal of the National Crises Management Center [3,4]. Vigorous efforts were exerted to detect and track cases and contacts by outbreak surveillance teams at the national and governorate levels in order to contain the spread of the virus and to isolate cases. The ultimate goal of Jordan was to flatten the disease spread curve in order to increase the capacity of the health system to absorb new cases [3,4].

In the UAE, the current widespread physical distancing and lockdown measures taken and the ramping up of testing have been successful in identifying new cases of COVID-19. However, the average number of new cases (from April to May) is estimated at 300-500 per day and rising [1,2,7]; it may still prove early for the country to ease its restriction measures. At this point, planning a cautious and responsive “exit strategy” is appropriate, but there remains a need for an even stronger capacity to test, retest, identify, quarantine, trace, and isolate contacts. In order to suppress transmission, public health and social measures should continue both at the individual and community levels. Individuals will need to maintain movement restriction measures at their own discretion, wear masks in public places, and maintain a 2-meter distance; international travel restrictions will continue to be implemented [1,2,7]. It is unknown how long this pandemic will continue, and the possibility of a surge in COVID-19 cases once restrictions are lifted is likely. It is advised that the government consider lifting restrictions when the number of new cases drops to 40-50 per day, with strict surveillance controls and 14-day intervals to identify the effects of loosening lockdown measures [1,2,7]. In reality, even the best plan may be insufficient, such as in the case of Singapore where lockdown measures were lifted after initial success and then reinstated due to a surge in cases [1,2,7]. Until effective pharmaceutical interventions (therapies and vaccines) are made widely available, the UAE will need to continue alternating between loosening and reinstating measures throughout this pandemic [1,2,7].

## Lockdown Lifting Overview

In an ideal situation, the requirements for lifting the lockdown would include the following:

1. Control the spread of the virus in a way that ensures a continuous reduction in new cases and a decrease in reproduction rate (R0) to less than one (ie, on average, each COVID-19–infected person may infect one other person or less over the most extended possible period) [8]
2. Preparedness of public health and curative services to contain all new cases and the contact spread chain, whether from a local source or for those who come from abroad, through the following measures [9]:
  The ability to epidemiologically detect suspected cases within 48 hours of the appearance of symptoms
  The ability to effectively isolate all diagnosed cases in hospitals or identified facilities
  The ability to detect, trace, quarantine, and monitor the close contacts of suspected or confirmed COVID-19 cases
3. The reduction of the potential spread of COVID-19 in congregated settings with a large number of people that are in close contact in the most vulnerable populations and areas such as nursing homes, nurseries, kindergartens, schools, universities, restaurants, religious or entertainment events (ie, minimizing outbreak risk in these settings)
4. The ability to manage evacuated returnees and those crossing the border (eg, shipments) to minimize the risk of spreading the epidemic (importation risk management)
5. The community and citizens should be aware of the measures to be taken when responding to the lockdown lifting; commitment and collaboration by identifying and reporting any new cases and cooperating to prevent the spread of the disease in large numbers is needed [8].

It is paramount to consider the notions of priority setting of public policies when it comes to lifting the lockdown via acknowledging the challenges, developing policy insights, and setting the policy direction [7,10].

## Strategies for Lifting the Public Sector Lockdown

Jordan and the UAE have already started the gradual lifting of the lockdown for private businesses and in some industries and local communities. The timing of movement regulations during the lockdown implementation is a critical element since access to public services and offices were restricted. As an effort to partially lift the lockdown measures, movement was allowed during specific times in both countries. During the lockdown, there were restrictions in terms of moving to and from public sector offices; only essential employees were permitted to move during the usual working hours of the public sector. In Jordan, the public sector lockdown exit began by permitting citizens to leave homes between 10 AM to 6 PM for reasons including visits to essential public offices (eg, to obtain medications for chronic patients). Meanwhile, in the UAE residents are allowed to leave their homes between 6 AM and 10 PM without a permit, which includes visits to essential public offices (eg, justice, foreign affairs, education, health, residency, infrastructures, municipalities, and judiciary).

It may not be necessary to wait for all the ideal requirements for lifting the lockdown to exist to open up public sector institutions. Accordingly, we provide our perspective on the most important strategies that may enable the opening process to achieve the overall goal of continuous reduction in the spread of the disease (case incidence) while gradually restoring normal life for society and the economy. The proposed strategies should be implemented slowly in stages, and an epidemic situational assessment should be completed at each stage to ensure there are no new cases detected. Once the stage proves successful, the next step can be implemented. If the epidemic situation were to worsen, it will be possible to resume lockdown measures.

### What Strategies Need to Be in Place?

The lockdown, which was initiated around mid-March and has lasted strictly until about the end of April, has caused much economic and social suffering, especially for the self-employed sector, big businesses, private clinics, and disadvantaged groups. Many have demanded a rapid lifting of the lockdown, which was considered and done gradually. However, the public sector, including health, higher education and vocational training, transport, etc, remained almost completely inaccessible. This viewpoint addresses the public health strategies and recommendations for the gradual lifting of the lockdown in these sectors.

**Figure 1:**
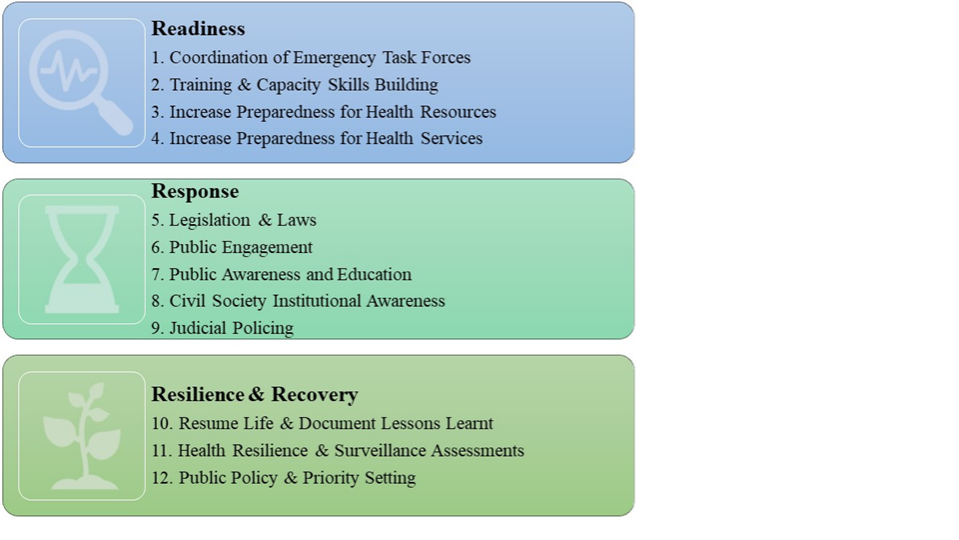
The 3 Rs of systematic approaches and leadership thinking required for lifting lockdowns.

The systematic approaches and leadership thinking required for lifting lockdowns in times of crisis include the three Rs: *Readiness*, *Responses*, and *Resilience & Recovery* (Figure 1). The first phase, *Readiness*, focuses on coordination, training, and preparedness; the second phase, *Responses*, refers to laws, engagement with the public, communities and civil society, and policing; and the third phase, *Resilience & Recovery*, involves documenting lessons learned and building resilience plans for the future. Below, we outline 12 recommendations and strategies for lifting the lockdowns with examples from Jordan and the UAE.

### Phase 1: Readiness

#### 1. Coordination of Emergency Task Forces

This step involves coordination between the local COVID-19 National Disaster Management Committee and the National Infection Control Committee in addition to the various ministerial government departments in order to manage and coordinate the opening process and public sector lockdown lifting nationally.

#### 2. Training and Capacity Skills Building

This involves the provision and training of sufficient numbers of COVID-19 investigation teams qualified to [9,11]:

- Conduct random COVID-19 testing of high-risk groups, in hot spots, and in different institutions that have a high population density (eg, nursing homes, institutional homes, refugee camps, labor accommodations, and labor camps)
- Scale up and continue contact tracing
- Carry out random testing of communities, industries, and institutions to detect asymptomatic cases
- Conduct sentinel surveillance of the workforce at different workplaces

These measures aim to ensure that the spread of the virus is under control and that there is an ability to detect and isolate new cases that may arise due to rapid reopening and to quarantine their contacts. It also will provide insight into how much herd immunity has been achieved. The most important criteria that should be monitored while applying this strategy are:

- The occurrence of an unexpected spike in new cases
- Continued reduction in the number of cases from unknown sources
- Rapid identification and control of hot spots and proper control of cases and their contacts

#### 3. Increase Preparedness for Health Resources

This involves raising the preparedness of available public laboratories and their technical and working staff and, when necessary, train and seek assistance from the private sector, retirees, and relevant, unemployed laboratory science graduates to accommodate the expected increasing number of COVID-19 polymerase chain reaction (PCR) diagnostic tests needed. The customary protocol requires conducting 152 screening tests per 100,000 people daily [12,13], which may be difficult to carry out in low-resource settings such as Jordan; however, over 140,000 COVID-19 tests have been completed with a ratio of 13,760 tests to 1 million people conducted throughout the crisis period [13]. In the UAE, there are over 1.5 million completed COVID-19 tests with a 158,000 to 1 million people test ratio [14-17]. However, these figures indicate the large effort and burden needed to apply this strategy, including:

- Providing large numbers of test kits
- Training the field infection investigation team staff on COVID-19 field sample collection protocol and other laboratory technicians on the procedures associated with running COVID-19 test in laboratories
- Ensuring safety and personal protection measures for staff

#### 4. Increase Preparedness for Health Services

This entails increasing the preparedness of hospitals and other curative service delivery posts by at least 20% to accommodate possible increases in the number of new cases requiring medical care at the national level. The construction of field hospitals is evident with a bed capacity of up to 3000 in the case of the UAE [7].

### Phase 2: Responses

#### 5. Legislation and Laws

This involves managing reopening at the provincial level by promoting the activation of the Decentralization Law or any relevant local government legislation. The experience of Jordan in managing the COVID-19 crisis in some governorates such as Irbid and Alaqaba was proven to be successful through contact tracing. This experience can be expanded to delegate the management of the opening-up measures at local levels to the local government and local executive boards. A thorough involvement of local community stakeholders who know which sectors have the highest priority to be opened is needed. Depending on the situation in each province, brigade, and locality in Jordan, and with continuous daily coordination at the national level, a more community-oriented lifting strategy may need to be achieved. Therefore, the roll-out strategy is to gradually increase the percentage of employees returning to public offices. Recently, in the UAE, a small number of public sector staff were allowed to work from the office, but this should not exceed 30% of the total number of employees [14].

#### 6. Public Engagement

The effective involvement of the communities, stakeholders, and individuals in the opening-up strategies across the public sectors is paramount. This strategy will encourage their serious buy-in commitment to reduce the number of new cases. They can be empowered to be actively involved in monitoring the case incidence by providing and encouraging innovative methods to report on suspected cases in person through private electronic platforms or to report on suspected cases in the workplace or among friends and family via other methods such as social media communication and different modes (event-based surveillance) and public information [16]. 

#### 7. Public Awareness and Education

It is paramount to effectively communicate with the public about the situation and policy measures in a timely manner to raise awareness levels. In addition, increasing health awareness by providing health education and public safety information to the public is needed. This responsibility falls on public, private, and civil society institutions alike (a holistic government-society approach) [18]. While it is expected that the leading role of raising health awareness of the disease and methods of social distancing lies on the shoulders of the Ministry of Health, all the public institutions that will open up have a significant role in raising awareness about the disease, and implementing social distancing measures [19] and personal protection measures using scientific models such as the Health Behavior Model. All institutions should be aware of their role in providing guidance on personal protection measures, social distancing, and in identifying the most critical symptoms of the disease and reporting suspected cases or their contacts, implementing self-isolation for a minimum of 14 days, and carrying out further tests to ensure negative results. For instance, citizens in Jordan and the UAE during the postlockdown period were required to not leave the house, with exceptions made for daily walking (Jordan) and exercise (UAE), grocery shopping, and other essential trips. Furthermore, there is evidence of coordinated public information campaigns (eg, across traditional and social media) [14,16]. Health awareness and promotion can be done in a comic way, a method that has proven to be effective in changing health behavior, especially in men (humorous persuasion) [20]. It can also be done by using religious symbols meaningful to some groups of society to reach most social classes, geographical areas, and working environments and institutions.

#### 8. Civil Society Institutional Awareness

This consists of involving civil society institutions in all phases of the opening process. The public sector is an essential partner in this crisis either in terms of raising awareness and field education for families and local communities or by involving them in infection field investigation measures. However, in the latter, they should receive adequate training in reporting suspected cases and in tracing their contacts. Civil society can also contribute to providing essential services to vulnerable populations in Jordan and the UAE, such as refugees, people with special needs, the elderly, orphanages, and other workers. According to the latest reports from the UAE’s Ministry of Health & Prevention [13], more than 97,645 workers from 31 labor accommodations were tested, and contact tracing for COVID-19 was implemented; a few of these cases were found to be positive.

#### 9. Judicial Policing

This involves activating the role of the “judicial police” and giving them the authority to refer establishments, institutions, or individuals who do not comply with personal protection measures and social distancing to the relevant authorities under the activated emergency legislation. Strict government policy adherence in Jordan and the UAE is vital, especially those related to school closures, workplace closures, cancellation of public events, restrictions on public gatherings, closures of public transport, stay-at-home requirements, general information campaigns, restrictions on internal movements, and international travel bans [16]. For instance, according to local UAE reports, Sharjah Police recently issued 3901 fines for violating movement restrictions, and in Dubai fines amounted to 52,000 for violating restrictions [21]. Such measures will make lifting the lockdown easier as they will ultimately reduce the case incidence and help the government to resume its activities gradually in a safe environment.

### Phase 3: Resilience & Recovery

#### 10. Resume Life and Document Lessons Learned

This step involves taking advantage of the comprehensive database found in some public and private institutions that show population data at the level of neighborhoods to document lessons to be learned [4]. In addition, it is essential to highlight how different services are distributed in each area, in addition to the resources available in local communities and at institutions, so that each area can function independently and facilitate the use of health and nonhealth services to detect and isolate cases, trace contacts [15], and quarantine suspected cases easily [7].

#### 11. Health Resilience and Surveillance Assessments

This strategy involves restarting the provision of public services at the national and local levels. This will decrease the burden on the secondary care level, which needs to be ready for a possible increase in the number of COVID-19 cases [19]. In the UAE, telehealth services have been implemented during the COVID-19 outbreak; such telehealth services should be continued in order to lessen the burden of health care services if confirmed cases were to increase [7].

#### 12. Public Policy and Priority Setting

This involves setting criteria for lifting the lockdown beginning with vital public sectors such as health and food security followed by other sectors in a gradual manner that provides enough time after reopening to detect any new or suspected cases and their contacts [7,9]. The standard strategy is to resume lockdown procedures if the epidemic situation worsens. The proposed criteria that may be used depends on:

- The contact intensity and density; that is, either high-risk exposure contacts who have spent 15 minutes or more in close proximity (≤2 meters) or in a closed environment; or low-risk exposure contacts who are still at risk but who have not been exposed to a confirmed case for as long;
- The number of persons or crowd number (contact number), which is given a value that ranges between high, medium, and low [7,21]; and 
- Crowd reduction (risk modifying likelihood ability), which describes the institution’s ability to introduce measures such as spatial or physical distancing into a space that will control the number of people in contact within a distance of 2 meters. These criteria are also given a value that ranges between high, medium, or low.

Based on the assessment of these three criteria and consensus on the value, vital sectors can initiate reopening while active monitoring and case detection are continuously exercised and kept in line with the criteria mentioned above.

## The Way Forward

It is critical to reinforce the notions of priority setting of public policies and public health strategies when it comes to lockdown lifting nationwide and keep in mind the challenges that may lie ahead. Thus, it is important to have mobilized teams in place for developing policy insights and setting the policy direction [22] and execution. Once the lockdown is lifted, the way forward for Jordan as well as the UAE would be to have policies in place to increase public awareness and implementation of the most important public health measures with a focus on physical distancing and personal protective equipment. Both countries should also increase the number of PCR tests, particularly for vulnerable populations and areas, and strengthen contact tracing measures. Preparation in terms of health system secondary care facilities, equipment, and supplies, in addition to an adequate number of trained and skilled health workers, is a must in light of a potential COVID-19 resurgence.

## Abbreviations

COVID-19: coronavirus disease
PCR: polymerase chain reaction
R0: reproduction rate
SARS-CoV-2: severe acute respiratory syndrome coronavirus 2
UAE: United Arab Emirates
WHO: World Health Organization
